# A Rare Case Presentation of Intraoral Palatal Myoepithelioma

**DOI:** 10.3390/reports8040196

**Published:** 2025-10-03

**Authors:** Abdullah Saeidi, Albraa Alolayan, Hattan Zaki, Emad Essa, Shadi Alzahrani, Wamiq Fareed, Shadia Elsayed

**Affiliations:** 1 Department of Oral and Maxillofacial Diagnostic Sciences, College of Dentistry, Taibah University, Madinah 42353, Kingdom of Saudi Arabia; abolayan@taibahu.edu.sa (A.A.); hzaki@taibahu.edu.sa (H.Z.); eeessa@taibahu.edu.sa (E.E.); ssayed@taibahu.edu.sa (S.E.); 2 Health and Life Research Center, Taibah University, Madinah 42353, Kingdom of Saudi Arabia; 3 Department of Oral and Maxillofacial Surgery, Faculty of Dentistry, Tanta University, Tanta 31527, Egypt; 4 Department of Oral and Maxillofacial Surgery, King Abdulaziz University, Jeddah 21589, Kingdom of Saudi Arabia; saalzahrani12@kau.edu.sa; 5 Department of Oral and Maxillofacial Surgery, College of Dentistry and Pharmacy, Buraydah Private Colleges, Buraydah, Al Qassim 51418, Kingdom of Saudi Arabia; wamiq.fareed@bpc.edu.sa

**Keywords:** myoepithelioma, salivary gland, benign tumor, palate, oral lesion, excisional biopsy, smoking

## Abstract

**Background and Clinical Significance:** Palatal swellings may originate from various pathological disorders. These swellings may include congenital or acquired factors. The posterior hard palate, which contains many minor salivary glands, is a common site for such swellings. **Case Presentation:** We present a rare case of intraoral palatal myoepithelioma in a 45-year-old Egyptian male with a significant history of smoking. Detailed clinical, radiographic, and operative findings are discussed alongside histopathological evaluation, surgical management, and postoperative outcomes. This case highlights the importance of considering myoepithelioma lesions in the differential diagnosis of posterior palatal swelling. **Conclusions**: Palatal myoepithelioma is a rare but important benign salivary gland tumor that may resemble multiple other intraoral lesions. A complete clinical, radiographic, and histological investigation is required for a definitive diagnosis. Complete surgical excision achieved a favorable outcome. Increased awareness and reporting of this unusual pathology are critical for deepening knowledge and guiding clinical decisions.

## 1. Introduction and Clinical Significance

Myoepitheliomas (MEs) are rare benign salivary gland tumors, accounting for 1.0–1.5% of all salivary gland neoplasms [1,2]. These tumors can occur in specific locations, primarily affecting the head and neck region, and most commonly occur in the parotid gland, vulva, and oral cavity, with higher prevalence in palatal minor soft salivary gland sites at 2.2–5.7% [3,4,5,6,7,8,9]. Other locations include the retromolar area, nasal cavity, larynx, and soft tissue (in rare extra-salivary cases) [10,11,12]. Most ME case reports that have been published were benign cases and had salivary gland origins. A smaller percentage reports more aggressive clinical cases of malignant MEs, also known as myoepithelial carcinomas [13]. These tumors are asymptomatic, slowly growing masses that can persist for months to years even in malignant variants [14,15,16]. Clinical presentation is non-specific; patients often report painless swelling, with some cases involving the soft tissues of the limbs and trunk [12,17]. In rare cases, ulceration, fast development, recurrence, or invasion has occurred, especially in malignant forms. Histologically, MEs are well-circumscribed, encapsulated tumors composed of myoepithelial cells and measuring 1–5 cm in diameter, which are usually associated with salivary gland ducts and arranged in various patterns, such as spindled and/or myxoid or plasmacytoid [2,15,16], and display diverse growth patterns and cell types, including spindle, hyaline, epithelioid, and clear cells [18]. Dentists frequently face challenges in diagnosing these swellings, necessitating careful consideration of various differential diagnoses because of their histological diversity and overlapping features [19]. Fine-needle aspiration presents diagnostic challenges, necessitating clarification of cytologic criteria [9]. Complete surgical removal is the preferred treatment, and benign forms behave similarly to pleomorphic adenomas (PAs) in terms of local expansion and recurrence potential [20].

When analyzing intraoral palatal swellings, the differential diagnosis is broad because both salivary and non-salivary gland lesions can be present [21,22]. We present a rare case of intraoral palatal ME in a middle-aged Egyptian male patient in order to explicate its diagnostic challenges, surgical planning, and histopathological characteristics, which are critical for definitive diagnosis and treatment planning. Increased awareness and reporting of such unusual pathologies are critical for deepening knowledge and guiding clinical decisions.

## 2. Case Presentation 

### 2.1. Clicical History 

A 45-year-old Egyptian male patient presented to the outpatient clinic of Taibah University Dental Hospital complaining of a painless posterior palatal swelling, progressively enlarging over the past 6 months. His medical history was unremarkable, and he was a heavy smoker for >20 years, with no alcohol or drug abuse.

### 2.2. Clinical Findings

**Figure 1:**
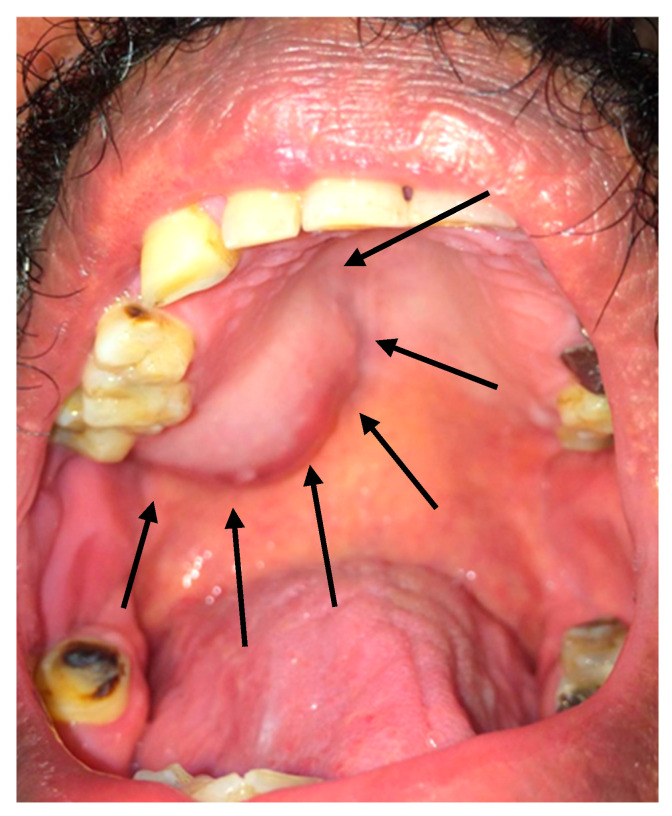
Intraoral photograph showing sessile firm unilateral posterior palatal swelling near the soft palate with normal colored mucosa.

Intraoral inspection and clinical intraoral examination revealed a firm, well-circumscribed, non-painful swelling located on the left posterior hard palate. The lesion measured approximately 2.5 × 2.0 cm and showed no ulceration of the overlying mucosa (Figure 1). The surrounding posterior dentition was sound and vital. Extraoral examination revealed no palpable cervical lymphadenopathy.

### 2.3. Radiographic Examination

**Figure 2:**
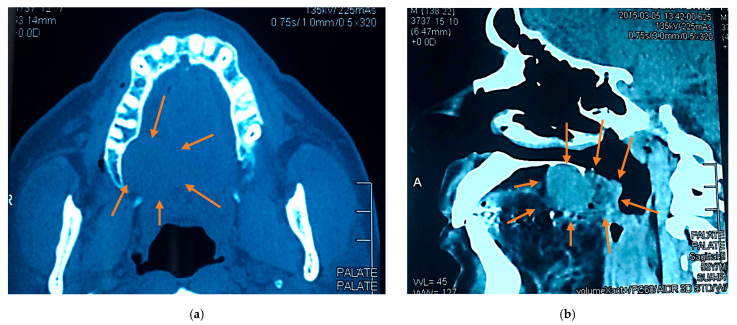
Axial (**a**) and sagittal (**b**) cone beam computed tomogram views showing a well-defined expansile soft tissue lesion at the posterior hard palate extending to the anterior soft palate; no resorption of the adjacent bone was observed.

Cone-beam computed tomograms showed a localized soft tissue mass encroaching on the alveolar bone without evidence of bony invasion or maxillary sinus involvement (Figure 2).

### 2.4. Differential Diagnosis

The overlying mucosa appeared intact and normal in color, suggesting that the lesion was likely submucosal in origin rather than inflammatory or ulcerative. The smooth surface and lack of discoloration or ulceration reduced the possibility of traumatic process, so the differential diagnosis might include benign salivary gland tumor, palatal cyst, palatal abscess (early stage), mucocele or mucous retention cyst, lymphoma or other soft tissue neoplasm, torus or bony exostosis, soft tissue hyperplasia (reactive lesion), or low-grade malignant salivary gland tumor.

### 2.5. Surgical Findings

**Figure 3:**
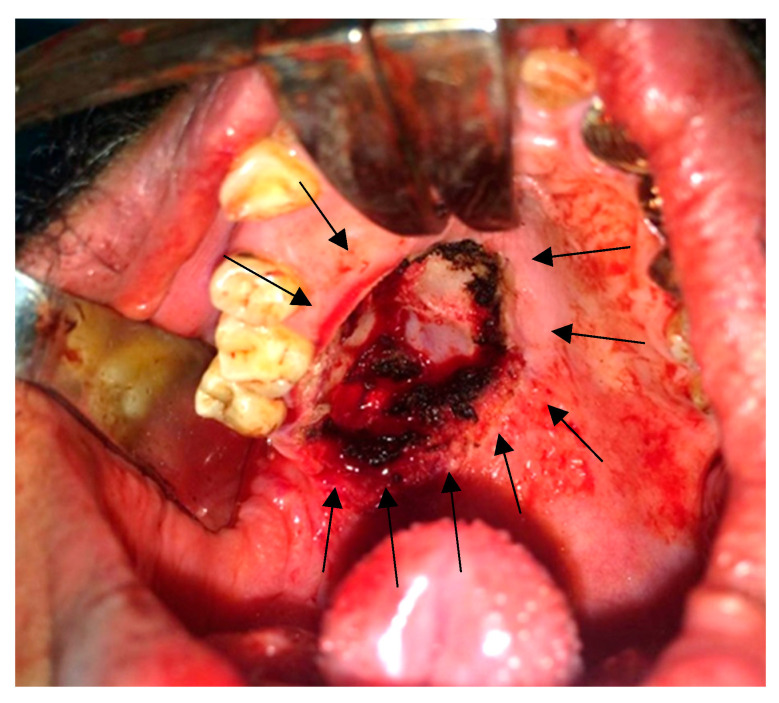
Intraoral photograph showing the surgical excision of the posterior palatal lesion with safety margin approach; the palatal bone was clear and intact, and hemostasis was achieved.

After we discussed the treatment approach to the disease with the patient and obtained informed consent for surgery under general anesthesia with nasal intubation, the patient underwent an excisional biopsy using an intraoral approach. A mouth gag and cheek retractors were used for exposure. A mucosal incision was made around the lesion (as indicated by the preoperative surgical markings with violet ink), and the tumor was excised completely. Hemostasis was achieved, and closure was done with absorbable sutures (Figure 3). The excisional biopsy was maintained in 10% formalin and sent to the histopathological department.

### 2.6. Histopathology

**Figure 4:**
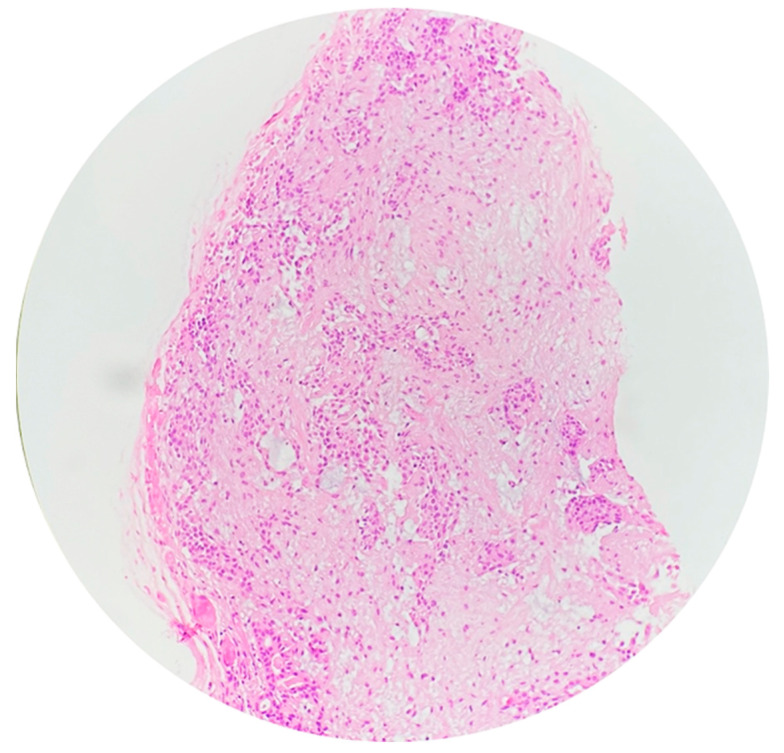
Microscopic examination shows nests and islands of plasmacytoid myoepithelial cells scattered in a fibromyxoid stroma (hematoxylin and eosin stain).

Microscopic examination revealed a neoplasm composed of uniform spindle-shaped and plasmacytoid myoepithelial cells arranged in a myxoid and hyalinized stroma. No ductal differentiation was noted. Mitotic activity was low, and there was no evidence of necrosis or infiltrative growth. Immunohistochemistry was positive for S-100, smooth muscle actin (SMA), and cytokeratin, confirming the diagnosis of benign ME (Figure 4).

### 2.7. Postoperative Course

**Figure 5:**
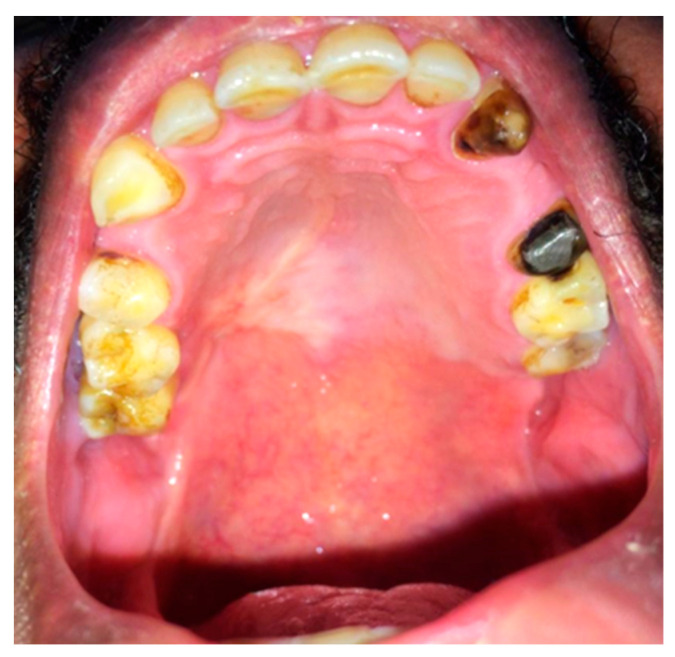
Intraoral photograph showing 1-month soft tissue healing post excision with secondary epithelization, without any signs of infection or dehiscence.

The patient recovered uneventfully via secondary healing with no evidence of recurrence at the 6-month follow-up. Smoking cessation was strongly advised, and the patient was referred for long-term follow-up (Figure 5).

## 3. Discussion

MEs are rare benign tumors arising primarily from salivary glands [23,24]. These tumors consist predominantly of myoepithelial cell proliferation and require careful diagnostic differentiation from other salivary gland neoplasms. The most significant distinction is between ME and PA, which are both slow-growing, painless palatal masses that may contain plasmacytoid/myoepithelial cells. However, PA has a biphasic epithelial-myoepithelial pattern with characteristic chondromyxoid stroma, whereas ME is made up almost entirely of myoepithelial cells, with few or no ductal elements. ME is well circumscribed and lacks infiltrative behavior when compared to low-grade malignant salivary gland tumors, adenoid cystic carcinoma, and polymorphous adenocarcinoma, which exhibit perineural invasion and infiltrative borders, whereas ME does not; mucoepidermoid carcinoma exhibits mucous and epidermoid cell differentiation with cystic change, which ME does not. Thus, rigorous histological examination, particularly the recognition of stroma type, ductal proportion, and development pattern, is required to distinguish ME from PA and low-grade malignant tumors [15].

Histologically, benign MEs lack specific diagnostic features in cytology and display various cell types and growth patterns, with a myxoid or hyaline stroma and absence of ductal structures [15,23,25]. Epithelial–myoepithelial carcinomas exhibit a characteristic biphasic tubular histology and can show various morphological changes, such as ancient change and sebaceous differentiation [26]. As shown in the present case, the advent of immunohistochemistry has been crucial for diagnostic confirmation, with positive staining for epithelial markers, S100 protein, and cytokeratin [4,27].

The tumors typically express α-SMA and p63 while lacking Ki67 expression [23], with positive reactions to vimentin, cytokeratin, and actin [2,15]. Recent molecular studies have identified specific signatures that can aid in accurate diagnosis and differentiation [27]. Additionally, immunohistochemistry has improved diagnosis by highlighting the pattern of these tumors. While they are generally low-grade, factors such as margin behavior, angiolymphatic invasions, and myoepithelial anaplasia can predict recurrence in epithelial-myoepithelial carcinomas [26].

While most cases are benign, these tumors can exhibit both benign and malignant variants. The latter are very rare but have been reported and classified as high-grade, especially in long-standing or recurrent cases [5,17]. The prevalence of oral cancer in Saudi Arabia is considered high according to Basha et al. (2019) [28]; however, the specific prevalence of ME is not mentioned in published studies [28]. The incidence of myoepithelial carcinoma is higher in adults and occurs across a wide age range (14–81 years), with no significant gender distribution or racial differences, affecting both sexes equally [29].

Recent studies have found a strong link between tobacco use and various salivary gland neoplasms, particularly Warthin’s tumor rather than ME, though not directly causative. Multiple investigations have found that a high percentage (94–97.5%) of patients with Warthin’s tumor had a history of smoking compared to significantly lower rates in patients with other tumor types [30,31]. Similarly, Yu et al. (1995) report a 96.9% smoking prevalence in Warthin’s tumor patients compared to 25.5% in controls [32]. Barbosa et al. (2022) report a single case of ME in a 49-year-old male with a 34-pack-year smoking history but state that this was the patient’s only risk factor without demonstrating a causative association [33]. Additionally, a population study in Wales revealed a significant correlation between higher smoking levels and epithelial-myoepithelial cancer incidence [14]. Our patient’s heavy smoking history may be considered a risk factor influencing tumor development or growth kinetics, warranting further study of environmental and lifestyle correlations. These findings collectively support a strong association between cigarette smoking and certain salivary gland neoplasms, warranting further investigation into this relationship. These studies, however, do not demonstrate a relationship between smoking and ME in particular, because they focus on other salivary gland cancers.

Management typically involves conservative complete surgical resection with a safe clear margin, which remains the primary preferred treatment for both benign and malignant forms of this lesion. Wide excision is indicated to decrease the risk of recurrence [27]. This conservative surgical excision is generally curative for benign cases, and prognosis is generally favorable, with low recurrence rates in malignant cases [14]. However, some soft tissue MEs may exhibit unpredictable behavior, with rare instances of metastasis reported [17]. Bhardwaj et al. describe a case of ME of the small salivary gland of the palate in a 23-year-old patient and the successful excision of the tumor [34], and Patankar et al. report the presence of small salivary glands in the palate of a 9-year-old child [35]. Several studies recommend long-term clinical and radiographic follow-up, often for at least 3–5 years, to detect late recurrences, emphasizing the significance of continuous follow-up beyond the 6-month term with our patient.

While early and radical surgery is highly recommended for favorable outcomes in malignant cases, radiotherapy appears to be ineffective, and elective neck dissection is generally unnecessary due to infrequent cervical lymph node metastasis. However, these tumors have a high rate of distant metastasis and frequent recurrence, with prognosis correlating with their histologic appearance [5]. Due to the rarity of these tumors, management is often based on surgeons’ experience, and more studies are needed to develop evidence-based guidelines [4].

Oral mucosa palatal wounds have a powerful and rapid healing capacity compared to skin or other soft tissues. In the present case, palatal surgical site approximation was not possible, and so the wound was left to heal by secondary epithelization following lesion excision. This healing approach involves granulation tissue formation, fibroblast proliferation, angiogenesis, and subsequent epithelial coverage from the wound margins without the need for primary closure or flap graft [36]. It usually takes 1 month to cover areas of bare intraoral bone, which may be due to high vascularity, the superior regenerative potential of the oral mucosa, and protective salivary factors; therefore, palatal wounds can achieve satisfactory aesthetic and functional outcomes with secondary healing due to the oral mucosa’s natural healing efficiency.

## 4. Conclusions

Palatal ME is a rare but important benign salivary gland tumor that may resemble multiple other intraoral lesions. A complete clinical, radiographic, and histological investigation is required for a definitive diagnosis. Complete surgical excision achieved a favorable outcome. Future studies and clinical practice should emphasize the use of specific immunohistochemical markers to develop a definitive, accurate diagnosis and management of palatal ME.

## Data Availability

The original contributions presented in this study are included in the article. Further inquiries can be directed to the corresponding author.
